# Round the Hospitals

**Published:** 1920-06-05

**Authors:** 


### ROUND THE HOSPITALS.

At the Investiture held on May 27 in the Quadrangle of Buckingham Palace the King personally conferred decorations on the following members of the nursing services: Bar to the Royal Red Cross: Miss Rachel Cox-Davies, T.F.N.S. Royal Red Cross First Class: Mrs. Vera Scholtz, Q.A.I.M.N.S.; Miss Esther Isaac, Q.A.I.M.N.S.R. Royal Red Cross Second Class: Misses Jessie Cairns, Isabella Craig, Florence Lerman, Elizabeth Mellor, and Mildred Street, Q.A.I.M.N.S.R.; Misses Christina Carnegie, Elsie Chaplin, and Helen Smith, Mrs. Emily Harvard-Davis, T.F.N.S.; Misses Emily Edwards, Colette Parker, and Margaret Riddell, B.R.C.S.; Miss Mary Cochrane, and Mrs. Geraldine Gould, Civil and War Hospitals; Miss Florence Broome, who also received

**ROUND THE HOSPITALS—***(continued)*.

the Military Medal, Civil Hospital Reserve; Misses B. Murgatroyd and I. Patton, V.A.D. Queen Alexandra received all the above ladies, together with Miss A. B. Smith, R.R.C., at Marlborough House after the Investiture. The nursing sisters were much gratified at Her Majesty's kindness in receiving them, notwithstanding the fact that she is still unable to make other engagements. We learn with much satisfaction that the Queen continues to make satisfactory progress.

The Queen paid an informal visit on May 31 to the City of London Maternity Hospital, City Road, of which she is Patron. Her Majesty, escorted by the Matron, Miss Greaves, and a distinguished company, visited every ward and was presented with a bouquet by one of the babies. The hospital was founded in 1750 during a wave of alarm at the scarcity of able-bodied men for the Army. It has never been more efficiently managed or more valuable to the community than at this moment.

The scheme of working hours for nurses suggested by the College of Nursing for inclusion in a Special Order in the Hours of Employment Bill (see p. 262) has two great merits. It includes all who are in direct connection with the nursing of the sick, except maternity nurses, and it deals with the question of hours in a thoroughly practical manner. The fifty-six-hour week, taken over a period of four weeks, with no one day's work to exceed ten hours, is a workable proposition. It has been tried with success in every department of nursing. The legal obligation on all employers to respect its observance would inflict hardship on none. Its enforcement would bring harmony into the nurse's life, and sweep away unmeasured trials from the lot of thousands of uncomplaining souls. The fifty-six-hour week does not exclude the possibility of a day's rest in seven. To fit fifty-six hours of work into the compass of six days nurses would work nine hours on four days and ten on two. Since the average hours per week in the case of institutions may be spread over a term of four weeks, there is considerable liberty of choice as to the disposition of off-duty time. A week-end once a month may be preferred to a weekly day. For private nurses the average is to be spread over a period of fourteen days. In both cases allowance is made for overtime, which is to be compensated by the same number of hours given as extra off-duty during the nurse's normal working hours. Those who regret that a fifty-six-hour week has been agreed to in lieu of a forty-eight-hour week must remember that any limitation by law of a nurse's working hours is a remarkable innovation, that the Bill merely imposes an outside limit to the demands which may be made on nurses' endurance, and that it will be greatly to the interest of institutions to attract a high class of probationer by lessening the maximum working hours. The drafting of the third paragraph of the Special Order suggested by the College of Nursing is extremely obscure, however.

The Florence Nightingale Medals, initiated by the International Red Cross at Geneva in 1912, will, under normal circumstances, be awarded annually to six nursing sisters who have rendered exceptional service. Some months ago we were requested to invite members of the nursing services to nominate sisters whose war services were exceptionally distinguished, and it is now announced that the following, whose names were submitted by the British Red Cross Society, are recipients of the first medals: Mrs. John Lambert, of the Royal Naval Nursing Reserve, for services in 1915 and 1916, especially on the hospital ship *Rewa*, at Gallipoli; Miss Beatrice Isabel Jones, R.R.C., C.B.E., Matron, Q.A.I.M.N.S., for services in Mesopotamia since 1916; Miss Gladys Laura White (Sister), B.R.C.S., for service, 1915 to 1918, especially at No. 9 B.R.C.S. Hospital at St. Omer (No. 36 Casualty Clearing Station); Miss Kate Maxey, R.R.C., M.M., Sister, T.F.N.S., for services from 1914 to 1918, especially at No. 58 Casualty Clearing Station; Miss Gertrude Mary Wilton Smith, Q.A.I.M.N.S., for services as Sister-in-Charge of Anglo-French Hospital Train No. 7 and No. 3 Casualty Clearing Station, France; Miss Margaret Clotilde Macdonald, R.R.C., Matron-in-Chief of the Canadian Army Nursing Service; Miss Lucy Minchin, Nursing Sister of the British Army in India and Mesopotamia; Miss Hester Maclean, R.R.C., Matron-in-Chief, New Zealand Army Nursing Service; Mrs. E. R. Creagh, O.B.E., R.R.C., Matron-in-Chief, South African Military Nursing Service. Forty-two medals have been awarded altogether, no distribution having been made during the war. The medal ranks as one of the most coveted distinctions of the world's nursing service, and our cordial congratulations are offered to the chosen few.

Sir E. Napier Burnett has been greatly impressed by the general shortage of probationers in the course of his preliminary survey of hospitals made as Director of Hospital Services to the British Red Cross Society. He ascribes it partly to the competition of other professions and partly to a spirit of revolt against long hours of drudgery, and blames the hospital authorities and the community for neglecting the social welfare and general training of the nurses. His suggestion that the Ministry of Health or the Board of Education might make grants to hospitals for the training of nurses is likely to be well received by those whom it proposes to benefit. Such grants, apportioned partly to the hospital and partly to supplement salaries, would enable the training of nurses to be carried on under greatly improved conditions. As usual, the good are confounded with the bad in public estimation, and institutions affording a highly advantageous course under comfortable conditions are banned because the probationer's lot is hard in others.

The establishment of primary and secondary health centres, as recommended by the Medical Consultative Council, and outlined in our last issue, would materially affect the interests of nurses in

**ROUND THE HOSPITALS**—(*continued*).

certain directions. More particularly it would redeem the nursing in cottage hospitals, which are marked out as suitable bases for the formation of primary health centres, from the many impediments to efficiency under which they now labour. The proposal that these little institutions, often unmanageable from the nursing point of view, should expand to provide communal and preventive services, pre-natal care, child welfare, physical culture, and so on, predicates an adequate nursing staff, and in areas where there is no efficient district nursing or midwifery service a visiting nursing staff would necessarily find a place also. The health-centre scheme, in fact, shadows a demand, not so much for more probationers as for more and ever better trained nursing sisters. In this it would prove a much-needed stimulus to the training-schools.

---

The Consultative Council of Medical and Allied Services, to whose report our correspondent in the Editor's Letter-Box (p. 259) refers, does not include a trained nurse, despite the representations made by the College of Nursing at the time of the Council's formation and since. The Federation of Medical and Allied Services, on which the College is represented, recently called a conference of nursing associations, which passed the following resolution which has been sent to the Minister of Health, "That there should be on the Consultative Council of Medical and Allied Services a qualified nurse, also being a certified midwife, who at the same time should represent the midwives, masseuses and sanitary inspectors." It is high time the Ministry of Health should realise the importance of the demand here voiced and should take steps to give effect to it.

---

The crisis in the affairs of the London Hospital is a matter of deep concern to all nurses in the Kingdom. With nearly a thousand beds, a staff of some 750 nurses, and an immense aggregation of special departments, it is the most impressive example of voluntary hospital work which the world has ever seen. That it should be driven to curtail its work would be a national calamity. And it will be a great blow to other voluntary hospitals in this country if it prove that the community is not able or not willing spontaneously to keep going so great a charity. If the benevolent are deaf to the necessities of the London they sound the knell of the voluntary system.

---

The Army Council has decided that pending the issue of general instructions regarding the cessation of Army of Occupation bonus, and the settlement of the new rates of pay at present under consideration, the Army of Occupation bonus will be issued to members of Q.A.I.M.N.S. (regulars). This bonus will not, however, be issuable for periods after April 30, 1920, to temporary nurses (Q.A.I.M.N.S., Reserve, T.F.N.S., V.A.D. Nursing Members, and special military probationers) unless they are in hospital or on sick leave, and are not in a position to sign the form of agreement to serve at home or abroad for a period not exceeding either one or two years.

---

The Romford Guardians are hard pressed for room in their Infirmary, and have been informed by Dr. Harvey, Ministry of Health Inspector, who attended a special meeting lately, that their first duty is to the children, whose requirements must be met, and their second to the nursing staff. Dr. Harvey very lucidly explained that it was more important properly to house the nurses within the institution than the private individual outside. He told the guardians, in answer to their inquiry, that the size of their nursing staff would shortly have to be increased so as to bring the average to one nurse to every six patients, and he stated that on his first appointment to his present post the proper number was considered to be 25 to 1.

---

Dr. R. Veitch, Medical Officer for Croydon, in his remarkable Presidential address, at the annual meeting of the Fever Nurses' Association, outlined a scheme for focussing into one central administrative unit, termed "The Nursing Institute," all the nursing services of a given area. This Institute would provide administrative offices and residential accommodation for the nurses, who would be available either for the private, visiting, or district work as required. The Institute would be closely linked with the various hospitals in the area and with its general public health work, so that nurses could be obtained in times of pressure for either service. One of the regulations suggested is that every nurse should once at least in every three years put in three months' work in hospital. A general superannuation scheme is forecasted, as well as an adequate salary and proper working conditions for the nurses. Unfortunately, Dr. Veitch has no sure leading on the subject of finance. This is one weak spot in his scheme which would be exceedingly costly. Another weak point is that if nurses are to be available in sufficient numbers to cope with every emergency or epidemic, many more must be kept in residence than could find work under normal conditions.

---

The Health Committee of the Bradford Corporation has considered a report by the Medical Officer showing the number of cases attended by the municipal midwives during the last quarter and the cases booked for the ensuing quarter, and decided to appoint four additional midwives forthwith.

---

The Southwark Guardians are pressing forward the projected extension to their infirmary nurses' home, and have accepted a tender for its erection. The new wing will cost over £13,000 and the nurses' dining-room in the main building which is now quite inadequate for the staff is to be enlarged at the same time. These alterations will add very largely to the comfort of the existing staff and by enabling more probationers to be received for training will materially lighten the labours of all.

**ROUND THE HOSPITALS**—(*continued*).

We fear many of our readers may be feeling the pinch of rising rents, and will learn with some dismay the particulars disclosed in the new Rent Bill, whereby an increase of from 25 to 40 per cent. may be imposed by the landlord. Most nursing homes are protected under the provision that no increases can be claimed during the currency of any lease or agreement, as the alterations required to fit ordinary dwellings for the reception of patients are so serious that few would carry them out without some security of tenancy. But the clauses relating to premiums are less satisfactory. No premium must be demanded for the grant, renewal, or continuance of a tenancy, except in the case of leases for seven years and upwards. This exception may hit the proprietor of going concerns with leases expiring very hard. Innumerable nurses living in unfurnished rooms or small houses have been put to great straits during the last year or two, and we fear many a home of this description has been broken up.

Miss F. E. Marquardt, matron for twenty-one years of the Camberwell Hospital, has tendered her resignation to the Camberwell Board of Guardians owing to ill-health. The Guardians have decided to grant her a superannuation allowance of £116 2s. 6d. a year.

A meeting of V.A.D. members was held at the Royal Automobile Club on May 28 to elect a representative and an advisory committee to supervise their interests in the disposal of their share of the United Services Fund. Lady Ampthill was in the chair, and she stated that the V.A.D. members had decided to associate themselves with the nursing services rather than with the society formed by the "Waacs," "Wrens," and "Wrafs." Lady Ampthill was unanimously elected as the V.A.D. representative. She warned the meeting against building their hopes on receiving too large a sum. They would probably be entitled to about 5s. per head. If the 40,000 members of the Red Cross who served in Auxiliary Hospitals were permitted to join in with the force of 32,000 V.A.D.s, they would undoubtedly be the largest body of women to receive it. Various suggestions were made for the disposal of the money to be allocated. One was the endowment of two hospital beds in memory of V.A.D.s who gave their lives in the war; another was the founding of a convalescent home and hostel. But in the end it was decided to form a Loan and Compassionate Fund. The money is to be invested and an advisory committee, which includes the names of Dame Beryl Oliver and Miss MacSwinney, was formed to deal with the management.

Mr. Frank Briant, M.P., Chairman of the Lambeth Board of Guardians, the Mayor of Lambeth, and the Rev. J. Bayfield Clark have interviewed the officials of the Ministry of Health in order to get their scheme for non-resident probationers sanctioned. As a result Dr. Addison suggests that the provision of hostels or boarding-houses would be the best way of meeting the housing difficulty. The Guardians must safeguard the nurses by providing the essentials of a healthy life, a reasonable amount of freedom and good training, with due regard to the requirements of the institution. Dr. Addison implies that other Boards of Guardians manage very well without resort to the living-out system, and the Lambeth Board has been stirred to inquire the names of the Poor Law institutions which are able to secure an adequate supply of probationers, with a view to copying their methods. It will be extremely interesting to learn the result. The St. Pancras Guardians have also petitioned for leave to employ non-resident probationers, and have received a similar reply.

The Central Midwives Board reports that there is a dearth of cases to form material for teaching purposes, and attributes this partly to the falling birth-rate and partly to changed conditions. The birth-rate appears, however, to be rising very suddenly indeed, and the changed conditions rather operate in favour of the student, for it is becoming very popular to lie-in at an institution, and the number of available beds for this purpose is undergoing a rapid increase. It is regrettable that women desirous of practising as midwives are crowded out at the training-centres by nurses who never intend to use their midwifery skill. Were all candidates for a certificate under compulsion to put in a period of midwifery practice after qualification it would (*a*) be very advantageous for such as accepted this condition; (*b*) considerably relieve the scarcity of practising midwives; (*c*) make more room in the schools. The lack of teaching material has been felt for a long time, and by medical students quite as much as by nurses. It can best be met by forming more training-centres with good maternity hostels.

The appeal for voluntary workers to aid in combating the influenza epidemic at Sheffield has been cordially responded to. Dr. Williams had over twenty volunteers for work at Lodgmoor Hospital, and they have now been three weeks at work, with the result that six empty wards have been opened. The matron, Miss Lewis, with the trained staff have successfully organised the nursing on these changed lines. Motor ambulances are fetching in victims of the disease from far and wide all day long, and as fast as a bed is vacated it is filled again. There is no doubt that many lives have been saved by this prompt action.

Great pleasure will be caused to a wide circle of our readers by the announcement that the annual gathering of "Old Nightingales" in connection with the Nightingale Training School is to be resumed after the long break during war years. The "At Home" will be held at St. Thomas's Hospital on Thursday, June 24, and any old Nightingale who fails to receive her invitation is requested to communicate her present address to the Matron in order that it may be forwarded to her. The occasion will be one of very special interest this year. It is the Diamond Jubilee, or Sixtieth Anniversary of the opening of the school.

